# Global trends in the incidence and mortality of asthma from 1990 to 2019: An age-period-cohort analysis using the global burden of disease study 2019

**DOI:** 10.3389/fpubh.2022.1036674

**Published:** 2022-11-22

**Authors:** Yu Cao, Sanqian Chen, Xiaoyun Chen, Wei Zou, Zhitao Liu, Yuhang Wu, Songbo Hu

**Affiliations:** ^1^School of Public Health, Jiangxi Provincial Key Laboratory of Preventive Medicine, Nanchang University, Nanchang, China; ^2^Jiangxi Cancer Hospital, Nanchang, China; ^3^Department of Epidemiology and Health Statistics, Xiangya School of Public Health, Central South University, Changsha, China

**Keywords:** asthma, incidence, mortality, global burden of disease, age-period-cohort analysis

## Abstract

**Background:**

Asthma is a major global health challenge. The global strategic management and prevention of asthma report has been published, but health system planning for asthma requires a careful assessment of asthma epidemiology. This study described the incidence and mortality of global asthma from 1990 to 2019.

**Methods:**

Based on data from the global burden of disease study (GBD) 2019, we present spatial and temporal trends in asthma incidence and mortality for the world and its 204 countries and territories from 1990 to 2019. Meanwhile, age-period-cohort analysis was used to explore factors influencing asthma incidence and mortality.

**Results:**

From 1990 to 2019, the incidence of asthma decreased from 601.20 per 1,00,000 to 477.92 per 1,00,000, and the mortality of asthma decreased from 8.60 per 1,00,000 to 5.96 per 1,00,000. High sociodemographic index (SDI) areas have higher age-standardised asthma incidence and low sociodemographic index areas have higher age-standardised asthma mortality. The age-period-cohort analysis results showed that the relative risk (RR) of incidence was high in children and the RR of mortality was high in elderly individuals. The RR of both asthma incidence and mortality showed a decreasing trend over time. The RR of asthma incidence in the recent birth cohort was higher than that in the previous birth cohort. The RR of asthma mortality continued to decline with the change in the birth cohort.

**Conclusions:**

Global asthma incidence and mortality decreased from 1990 to 2019. The decline in asthma incidence was mainly attributed to age effects and period effects, and the decline in asthma mortality was mainly attributed to period effects and cohort effects. Focusing on the risk of incidence in children and the risk of mortality in the elderly, promoting healthy lifestyles and controlling environmental risk factors can help to better control asthma.

## Introduction

Asthma is a heterogeneous disease usually characterised by chronic airway inflammation. It is defined by a history of respiratory symptoms such as wheezing, shortness of breath, chest tightness, and cough that vary over time and in intensity, together with variable expiratory airflow limitation (1). Asthma is a serious global health problem, and people of all ages worldwide are affected by this chronic respiratory disease. The global prevalence of asthma was estimated to be 3.33% in 2017, and asthma was the second leading cause of death among chronic respiratory diseases, with 457.01 thousand deaths due to asthma in 2017 (2). In addition to causing premature death, asthma is often associated with various comorbidities, such as allergic rhinitis, gastroesophageal reflux disease, obstructive sleep apnoea, and anxiety (3, 4), which seriously affects quality of life. In addition, asthma imposes an additional economic burden on patients and their families. In the United States alone, the total cost to society from asthma was estimated at $56 billion in 2007, including medical costs ($50.1 billion per year), lost productivity due to absenteeism and missed school days ($3.8 billion per year), and premature deaths ($2.1 billion per year) (5).

The Global Initiative for Asthma published its landmark Global Strategic Management and Prevention of Asthma report in 2014, which proposed to improve the lives of people with asthma around the world, raise public awareness of asthma, and help reduce asthma incidence and mortality by improving management and promoting the availability and accessibility of asthma treatment (6). The development of asthma-specific health services requires informative epidemiological estimates, including estimates of asthma incidence and mortality in specific regions and populations. Most previous analyses of asthma incidence and mortality have been limited to small data sets or localised areas. In our study, the results of previous global burden of disease study (GBD) studies on asthma were updated, and richer results were presented based on the latest estimates of asthma from the GBD 2019, combined with epidemiological models.

This study visualised global asthma incidence and mortality data by age, sex, year, and region for the period 1990–2019 to understand temporal and spatial trends in asthma. This study further analysed the contribution of age, period, and birth cohort effects to changes in asthma incidence and mortality over the past three decades through age-period-cohort analysis, which was essential to explore the factors affecting asthma incidence and mortality from a macroscopic perspective. Therefore, the results of the study may provide a scientific basis and data support for global asthma control efforts and provide some guidance for the development of prevention and control strategies to reduce the burden of asthma.

## Methods

### Data source

The GBD 2019 study was a comprehensive study conducted jointly with global collaborators that provided a comprehensive assessment of age-sex-all-cause and cause-specific incidence and mortality for 369 diseases and injuries in 204 countries and territories from 1990 to 2019. Asthma incidence was estimated based on the Bayesian meta-regression tool (DisMod-MR2.1), and asthma mortality was estimated according to the Standard Cause of Death Ensemble Modelling (CODEm). The original data used for estimating asthma mortality in GBD 2019 were derived from vital registration and surveillance data from the cause of death database, and the original data resources for estimating incidence included literature reviews, survey data, and certain national claims data (2). The detailed information on original data sources presented in supplementary materials. In GBD 2019, asthma was defined according to the diagnosis of asthma relied on self-reported wheezing and physician diagnosis over the past 12 months (Code 493 in the 9th revision of the International Classification of Diseases, J45 and J46 in the 10th revision) (2). The Socio-Demographic Index (SDI) is a composite indicator of the level of economic development of a region, with a higher index indicating a more developed economy. The SDI is the geometric mean of 0–1 indices of total fertility rate for those younger than 25 years old, mean education for those 15 years old and older, and lag-distributed income per capita (2). Different countries and territories were further divided into five regions, namely, low (0–0.455), low-middle (0.456–0.608), middle (0.609–0.690), high-middle (0.691–0.805), and high (0.806–1) SDI regions (2, 7). Additional detailed methodological information on asthma-related indicator estimation and modelling strategies in GBD 2019 was published elsewhere as supplementary material (2, 7, 8).

In this study, we extracted data on incidence, mortality, and its age-standardised rate of asthma in 204 countries and territories from 1990 to 2019 for analysis. The age distribution of the world population from the GBD 2019 study was used to standardise rates of incidence, mortality per 1,00,000 person-years of asthma (9). The 95% uncertainty intervals (UI) for every metric was based on the 25th and 975th ordered values of 1,000 draws of the posterior distribution (2).

### Statistical analysis

#### Descriptive analysis

This study reported global asthma incidence and mortality and their spatial and temporal trends from 1990 to 2019. We plotted asthma incidence and mortality from 1990 to 2019 to compare asthma across years. This study reported on asthma incidence and mortality in different SDI regions from 1990 to 2019 and provided a global view of asthma incidence and mortality, comparing differences in asthma incidence and mortality between countries and regions.

#### Age-period-cohort analysis

The age-period-cohort model is a commonly used statistical tool to extract information hidden in disease incidence and mortality. The model decomposes the study indicators into three dimensions, age, period, and birth cohort, and analyses the impact of age effect, period effect, and birth cohort effect on disease incidence and mortality (10). Age effect reflects the impact of age changes, including population ageing, on incidence or mortality. Period effect refers to the impact on incidence or mortality due to changes in certain objective factors over a period of time, such as disease screening practises, treatment and interventions. Cohort effect refers to the influence on incidence or mortality due to different degrees of exposure to disease risk factors among different birth cohorts. However, there is an exact linear relationship between age, period, and cohort, and the linear regression model cannot find a unique estimate of the effect of these three factors, which can be solved by the intrinsic estimator method; the methodological details are described in previous literature (11, 12).

When using the age-period-cohort model combined with the intrinsic estimator algorithm (APC-IE), it is required that the age group data and period group data have the same structure, so we divided the population aged 0–94 years into 19 age groups (0–4, 5–9,…, 89–94) with a group distance of 5 years. Then, to prevent temporal overlap of adjacent birth cohorts over the entire observed period from 1990 to 2019, time point values (1992, 1997,…, 2017) were used for analysis instead of the average of the 5-year periods. Birth cohort = period-age, so a total of 24 birth cohorts (1900–1904, 1905–1909,…, 2015–2019) were generated based on 6 period groups and 19 age groups.

Coefficients and 95% confidence intervals (CI) for age, period, and birth cohort were calculated in our study. The coefficients were then calculated to their exponential value [exp (coefficients)] that denoted the incidence and mortality relative risk (RR) of a particular age, period, or birth cohort relative to each average level (13). The average level is the average of the cohort, period, and age coefficients. When the RR value is greater than 1, it indicates that the factor has an effect of increasing the risk of asthma incidence or mortality. When the RR value is less than 1, it indicates that the factor has an effect on reducing the risk of asthma incidence or mortality. APC-IE analysis was performed through the Stata 12.0 software.

## Results

### Global, regional, and national trends in asthma incidence and mortality

**Figure 1:**
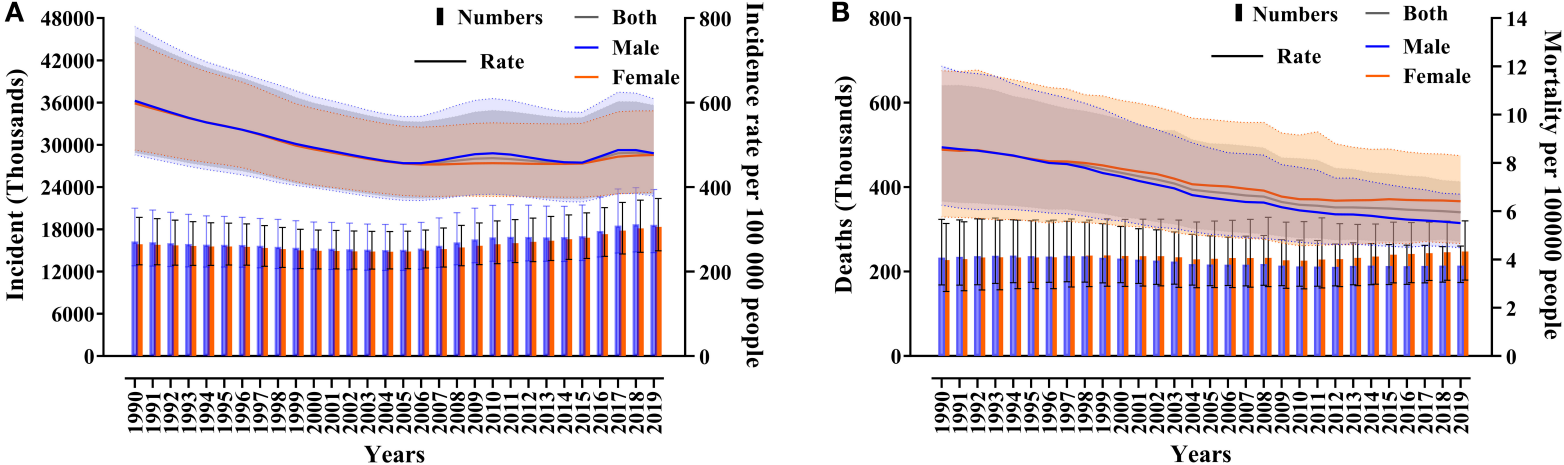
Global asthma incidents, incidence **(A)**, deaths, and mortality **(B)** from 1990 to 2019.

According to GBD 2019, the number of asthma incidents increased from 32163.21 (95% UI 25,752.79–40,513.13) thousand to 36,979.27 (95% UI 29,601.98–45,928.11) thousand from 1990 to 2019, an increase of 14.97% (95% UI 11.71–17.96). The incidence of asthma decreased from 601.20 per 1,00,000 (95% UI 481.37–757.28) to 477.92 per 1,00,000 (95% UI 382.58–593.58) (Figure 1A). There was no sex difference in the incidence of asthma. From 1990 to 2019, the number of asthma deaths increased from 460.01 per 1,00,000 (95% UI 342.62–599.60) thousand to 461.07 (95% UI 366.58–559.01) thousand, with little change. Asthma mortality decreased from 8.60 per 1,00,000 (95% UI 6.40–11.21) to 5.96 per 1,00,000 (95% UI 4.74–7.22), and asthma mortality declined more in males than females (Figure 1B).

**Figure 2:**
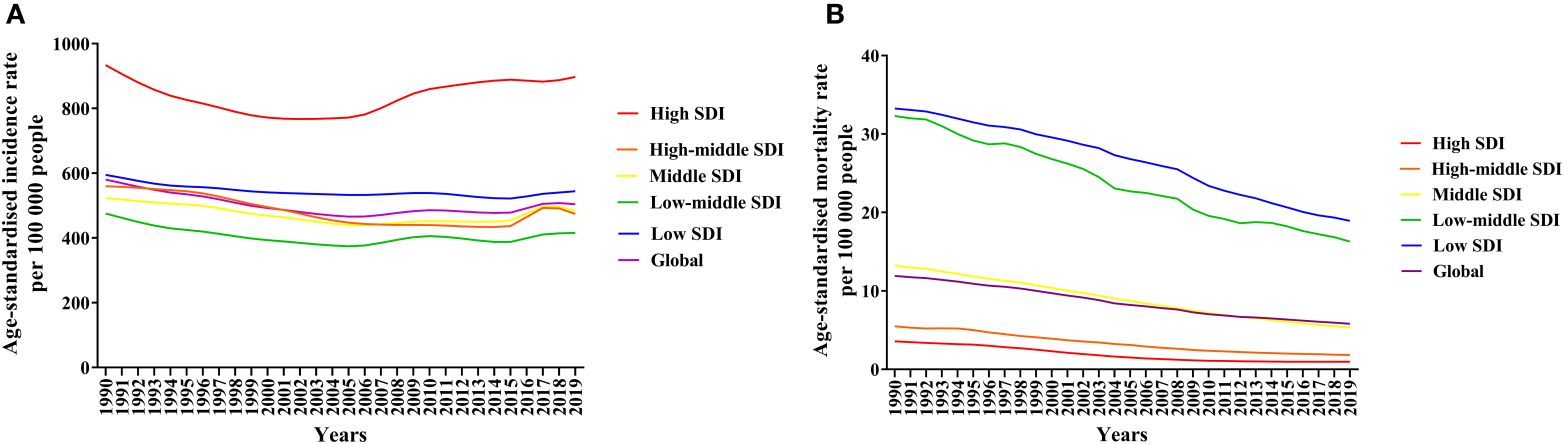
Global age-standardised incidence rate **(A)** and age-standardised mortality rate **(B)** of asthma by SDI regions, 1990 to 2019.

We analysed the age-standardised incidence rate and age-standardised mortality rate of asthma by SDI region. The age-standardised incidence of asthma in all SDI regions showed a fluctuating downwards trend between 1990 and 2019, with the highest age-standardised incidence of asthma in high SDI regions and little difference in other regions, which did not show a trend with SDI changes (Figure 2A). The age-standardised mortality rate of asthma showed a decreasing trend in all SDI regions, especially from low SDI regions to high SDI regions, where the age-standardised mortality rate of asthma decreased sequentially (Figure 2B).

**Figure 3:**
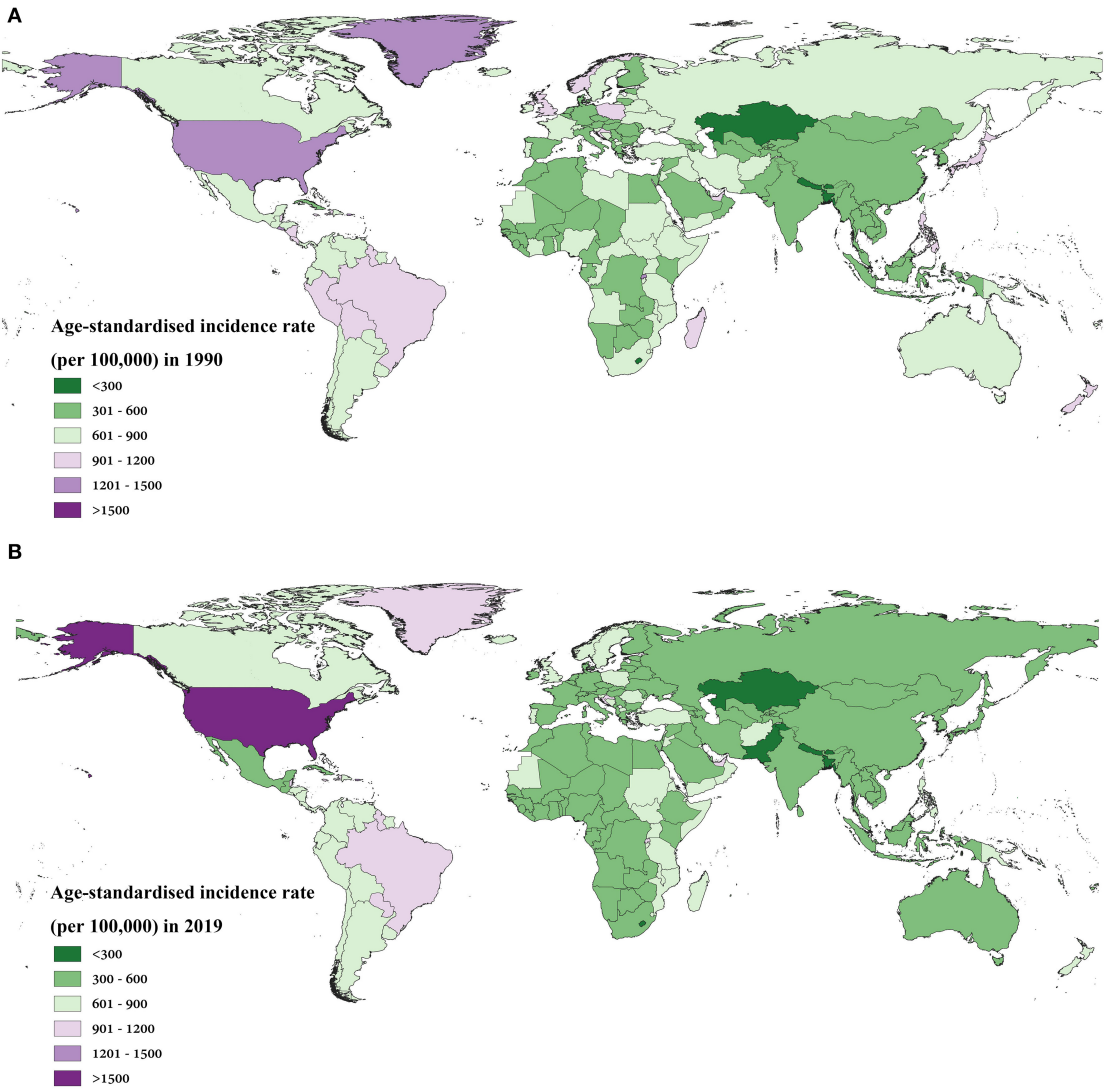
The age-standardised incidence rate in 1990 **(A)** and 2019 **(B)** for asthma in 204 countries and territories worldwide.

We further provided SDI values (Supplementary Table 1) by country and territory for 2019 and provided a global view of asthma incidence and mortality. The age-standardised incidence rate and age-standardised mortality rate of asthma varied widely between countries and territories. In 1990, the areas with higher age-standardised incidence rates of asthma were El Salvador, Rwanda, Puerto Rico, Greenland, and the United States of America, all over 1,200 per 1,00,000. In 2019, the age-standardised incidence rate of asthma exceeded 1,200 per 1,00,000 in Puerto Rico and the United States only, and the age-standardised incidence of asthma in the United States increased to 1,547.24 (95% UI 1,251.20–1,899.94) (Figure 3). The age-standardised asthma rate decreased in most countries and territories during the three decades, with some of the larger declines concentrated in Europe, Asia and Oceania (Supplementary Figure 1A).

**Figure 4:**
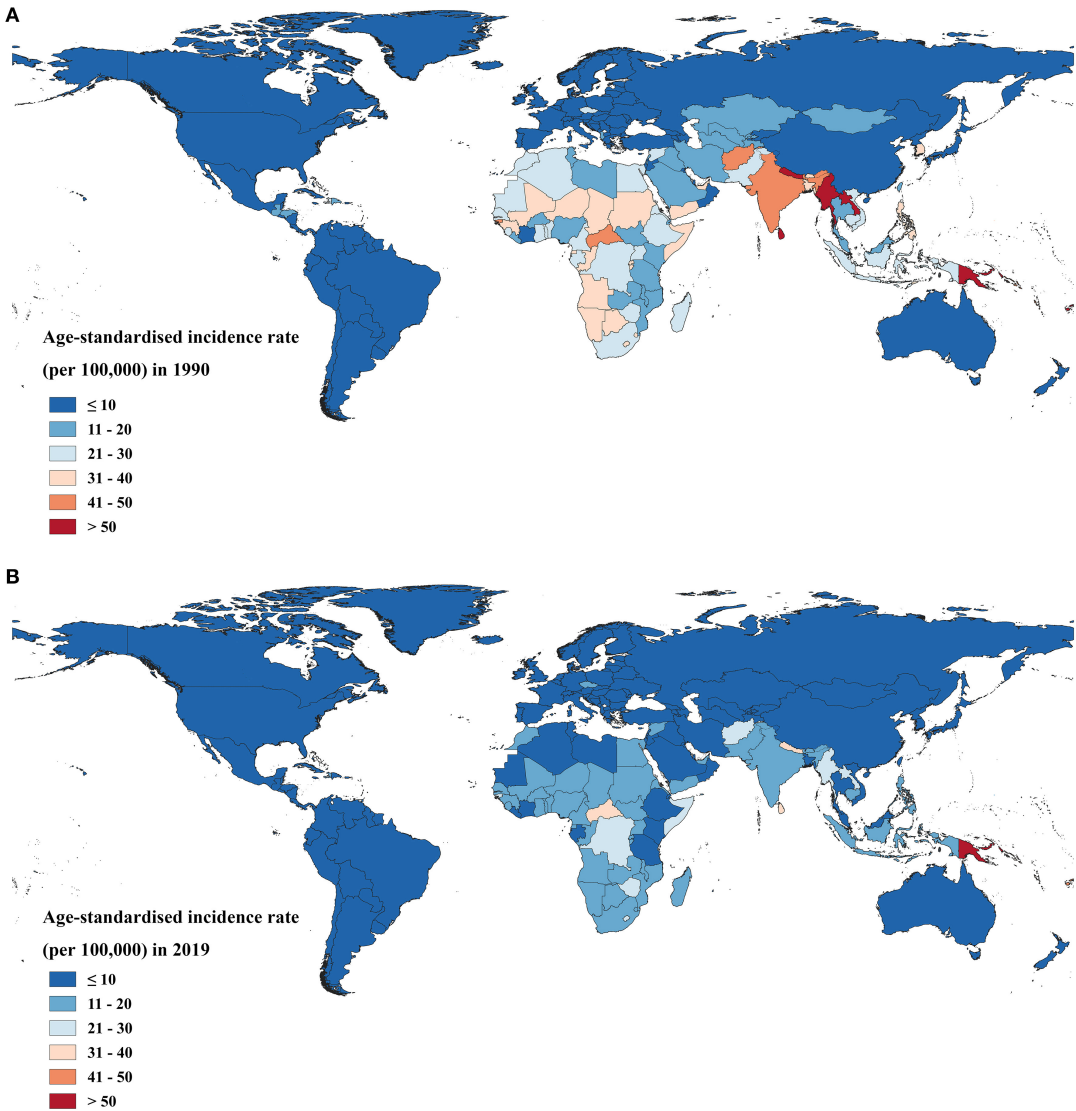
The age-standardised mortality rate in 1990 **(A)** and 2019 **(B)** for asthma in 204 countries and territories worldwide.

The asthma age-standardised mortality rate declined in all regions, with a decline of more than 50% in the vast majority of high-SDI and high-middle SDI countries and territories (Supplementary Figure 1B). The age-standardised mortality rates of asthma for Kiribati, Fiji, Sri Lanka, Myanmar, Papua New Guinea, Nepal, Federated States of Micronesia, Lao People's Democratic Republic, and Tuvalu were higher than 50 per 1,00,000 in 1990. By 2019, only Kiribati and Papua New Guinea had an asthma age-standardised mortality rate higher than 50 per 1,00,000 (Figure 4).

### Descriptive analysis of asthma incidence and mortality by age, period, and birth cohort groups

**Figure 5:**
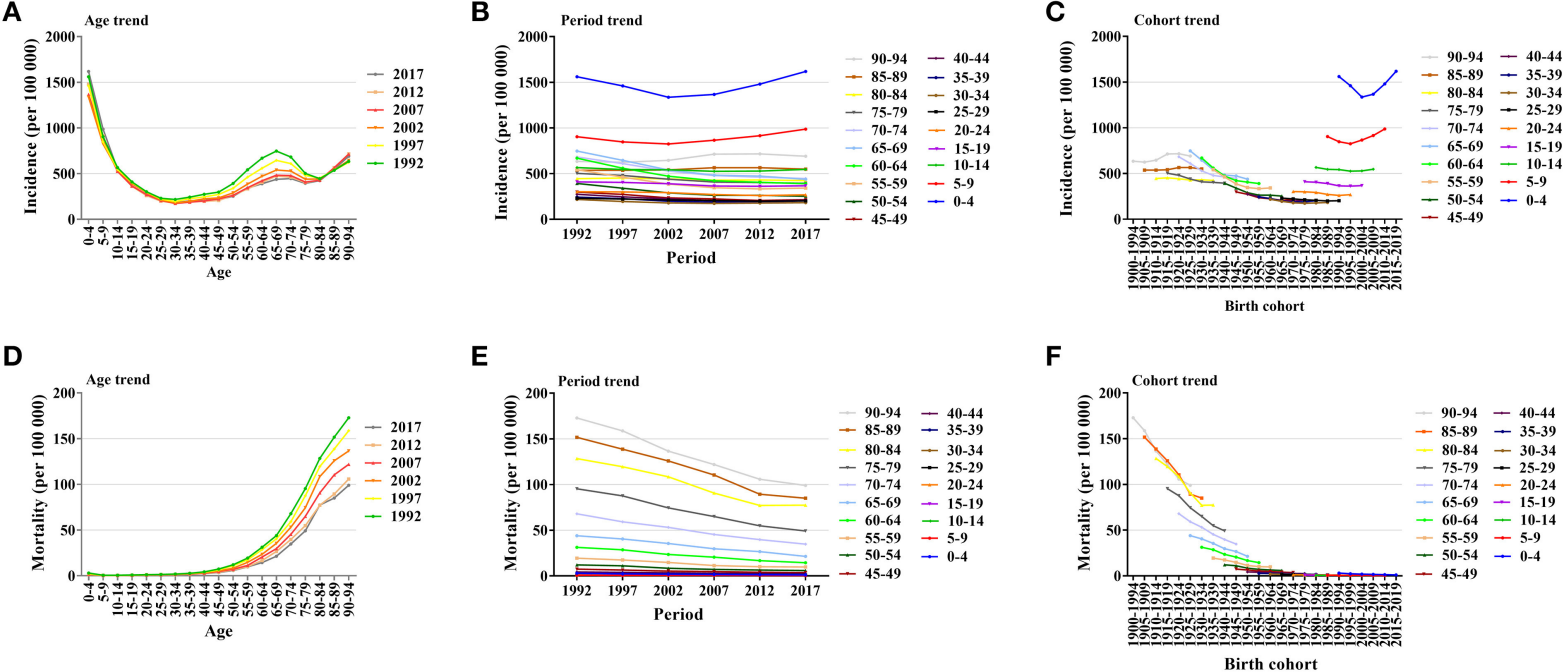
Global trends in asthma incidence and mortality. **(A)** Global trends in asthma incidence by age. **(B)** Global trends in asthma incidence by period. **(C)** Global trends in asthma incidence by birth cohort. **(D)** Global trends in asthma mortality by age. **(E)** Global trends in asthma mortality by period. **(F)** Global trends in asthma mortality by birth cohort.

Figure 5 illustrated global trends in asthma morbidity and mortality by age, period and birth group. The incidence of asthma first decreases and then increases with age within each period. Asthma incidence was highest in the 0–4 age group and lowest in the 30–34 age group. Asthma incidence does not change much over time in most age groups, with a slight increase in the 0–9 age group. The most recent birth cohort had the highest incidence of asthma. The mortality of asthma increased with age in all periods, with the highest mortality in the 90–94 age group. The mortality of asthma in all age groups decreased over time, with greater decreases in the higher age groups and fewer decreases in the lower age groups. There was a decreasing trend in asthma mortality across birth cohorts, indicating a relatively low risk of asthma mortality in the most recent birth cohort.

### Age-period-cohort effects on asthma incidence and mortality

The above trend analysis of age, period, and birth cohort in global asthma incidence and mortality cannot separate the interaction among the three factors. Furthermore, APC-IE analysis was needed. We calculated age, period, and birth cohort coefficients (Supplementary Tables 2, 3) for incidence and mortality based on the APC-IE analysis. Relative risks of asthma incidence and mortality were then calculated based on these coefficients to examine the effect of a single factor on the risk of asthma incidence and mortality.

### Age effects

**Figure 6:**
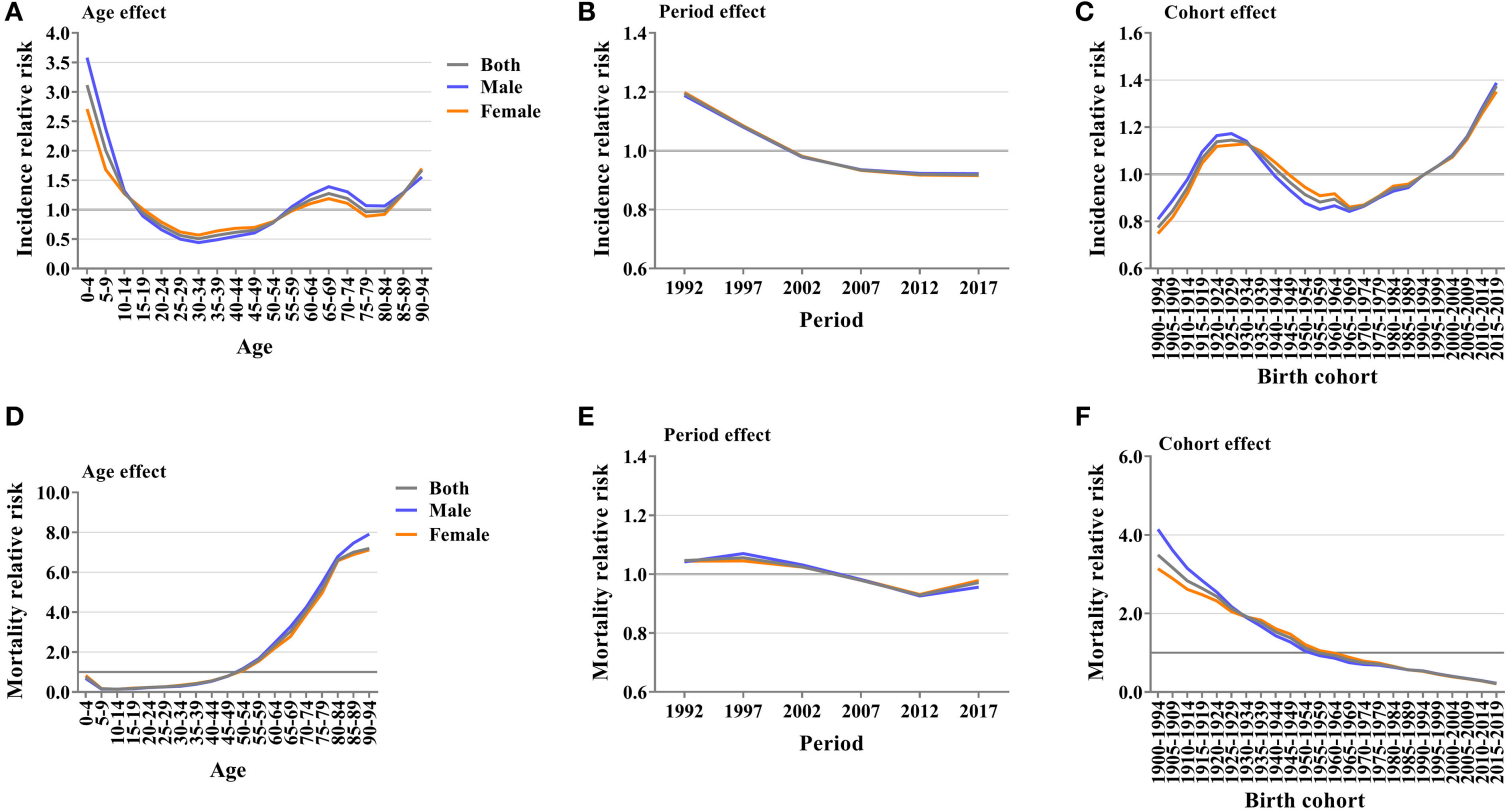
Relative risk of asthma incidence and mortality. **(A)** Relative risk of asthma incidence due to age effect. **(B)** Relative risk of asthma incidence due to period effect. **(C)** Relative risk of asthma incidence due to birth cohort effect. **(D)** Relative risk of asthma mortality due to age effect. **(E)** Relative risk of asthma mortality due to period effect. **(F)** Relative risk of asthma mortality due to birth cohort effect.

After controlling for periods and birth cohort effects, the RR of asthma incidence first decreased and then increased with age. Among all age groups, the highest and lowest RR values were found in the 0–4 age group and 30–34 age group, respectively. The RR of asthma incidence in the 0–4 age group was 3.11 times (95% CI 3.01–3.22) and in the 30–34 age group was 0.51 times (95% CI 0.48–0.54) compared to the mean. The RR of asthma incidence was higher in both the 0–14 years and most of the higher age groups (Figure 6A). The RR of mortality from asthma showed an overall trend of increasing with age. The RR of mortality from asthma in the 90–94 age group was 7.19 times (95% CI 5.13–10.09) higher than the average for all age groups, with the highest RR of mortality. The RR of mortality was greater than 1 for all age groups after the 50–54 age group, and the RR of mortality increased rapidly with age (Figure 6D).

### Period effects

After controlling for age and birth cohort effects, both the RR of asthma incidence and the RR of asthma mortality showed an overall decreasing trend over time, with period effects being relatively consistent in both sexes. From 1992 to 2017, the RR of asthma incidence decreased from 1.19 (95% CI 1.17–1.22) to 0.92 (95% CI 0.90–0.94), and the RR of asthma mortality decreased from 1.05 (95% CI 0.87–1.26) to 0.97 (95% CI 0.81–1.17) (Figures 6B,E).

### Cohort effects

After controlling for age and period effects, the RR of asthma incidence showed a trend of fluctuating variation with birth cohort changes. From the 1965–1969 birth cohort to the 2015–2019 birth cohort, the RR of asthma incidence increased consistently from 0.85 (95% CI 0.80–0.91) to 1.37 (95% CI 1.29–1.46). In the recent birth cohort, the RR of asthma incidence was higher than that in all previous birth cohorts (Figure 6C). The RR of asthma mortality showed a trend of decreasing with birth cohort change. From 1900–1904 to the 2015–2019 birth cohort, the RR of death decreased from 3.49 (95% CI 2.26–5.40) to 0.21 (95% CI 0.03–1.50). Cohort effects were relatively consistent in both sexes (Figure 6F).

In this study, APC-IE analysis of incidence and mortality in different SDI regions was performed, and the results of each region were generally consistent with the global results.

## Discussion

This study showed that global asthma incidence and mortality decreased from 1990 to 2019, but the number of incidents was still growing, and asthma remained one of the most prevalent chronic respiratory diseases. Global population growth may be responsible for the increased number of asthma incidents. The Global Initiative for Asthma has regularly published and annually updated global strategies for asthma management and prevention since 1993, providing guidelines for the management of asthma in many countries (6). The continued reduction in asthma incidence and mortality during this period may reflect the effectiveness of global asthma control outcomes. Although considerable progress has been made at the global level in reducing these indicators, worrisome trends were still observed in some regions.

This study found that the age-standardised incidence rate of asthma did not decrease with increasing SDI, and the age-standardised incidence rate was highest in high SDI regions. However, this finding differed from the GBD 2017 study results. Previous studies have shown that the age-standardised incidence rate of asthma remained low in high SDI and high-middle SDI regions, while the age-standardised incidence rate of asthma was highest in low SDI regions (14, 15). Between the GBD 2017 and GBD 2019 iterations, a methodological change led to an obvious difference in the incidence of asthma in high SDI regions. GBD 2019 added new data from the US and UK and adjusted the case definition in asthma-related estimates compared to GBD 2017; these changes reduced the bias of asthma estimates in high SDI regions (2). In addition, high SDI regions have better asthma surveillance and investigation systems, which also correspond to a high asthma detection rate.

It was almost universal that a low age-standardised mortality rate of asthma was observed in high SDI countries and territories, and a higher age-standardised mortality rate was observed in low SDI countries and territories. This finding suggested that inequalities in access to prevention and treatment services in lower socioeconomic groups may be a barrier to sustained improvement in asthma mortality. Underdiagnosis, misdiagnosis, and inadequate treatment in most low-income and low-middle-income countries led to considerable asthma incidents and deaths (16), which could have been avoided. Based on knowledge of asthma pathophysiology and clinical trial results, most asthma deaths in all age groups can be prevented by low-dose inhaled corticosteroids and other treatment strategies (17). Substantial evidence has proven the protective value of regularly inhaled corticosteroids, which significantly improved symptoms, reduced the frequency of asthma exacerbations, and significantly reduced the risk of death in patients with asthma (18, 19).

Age effects showed a high RR of asthma incidence in children and a high RR of asthma mortality in elderly individuals. Related studies have shown that asthma onset and severity are interlinked with genetic and environmental factors during pregnancy and childhood. Maternal smoking during pregnancy, premature birth, and childhood exposure to air pollution (ozone, sulphur dioxide, nitrogen oxide, and particulate matter) increase the risk of childhood asthma (5, 20, 21). Premature birth was the most dominant risk factor known to cause asthma (22). Asthma attacks in the elderly tend to be more severe and have a higher mortality rate than in children (23). Although the pathogenesis of asthma in the elderly was unknown, it was hypothesised that increased RR of asthma incidence in the elderly may be due to “normal” changes in airway structure and immune senescence that occurred with ageing. Immune senescence may increase susceptibility to respiratory infections and exacerbate underlying asthma in the elderly (24). Age effects have contributed to the decline in asthma incidence over the last three decades, which was associated with global demographic changes. From 1990 to 2019, the proportion of the population under the age of 14 decreased from 32.8 to 25.4%, and the proportion of the population aged 15–64 increased from 61.1 to 65.3% (8). A decrease in the proportion of the population at high RR of asthma incidence and an increase in the proportion of the population at low RR of asthma incidence would lead to a decrease in the incidence of asthma.

The dramatic increase in the RR of mortality of asthma in the elderly may be explained by the effects of comorbidities, such as malignant respiratory tumours, cardiovascular disease, and depression, which complicate treatment, reduce treatment adherence, and affect overall prognosis and survival (25–27). Age effects were not conducive to the reduction in asthma mortality. The proportion of the population over the age of 65 increased from 6.08 to 9.33%, and the increase in the proportion of people in the age group with a high RR of mortality would lead to an increase in the mortality of asthma.

The study found that the RR of asthma incidence and the RR of mortality decreased over time and were below the average risk after 2004. The period effect contributed to the decline in asthma incidence and mortality in the past three decades. From the perspective of the development process of global society, with the development of the economy, advance of medical technology, and improved living conditions, the RR of incidence and mortality of asthma should indeed show a decline over time. These declines might be the result of a long list of factors, including global or regional success in tobacco control measures, paired with reductions in environmental pollution in cities, more electric cars, cleaner working environments, better information and prevention about allergens, and more accurate diagnostics to distinguish asthma from other respiratory diseases (28).

Cohort effects in asthma showed that different birth cohorts had an impact on asthma incidence and mortality. Changes in the RR of incidence and mortality may reflect changes in exposure to a broad range of environmental risk factors or lifestyles within the same birth cohort. The RR increase for asthma incidence was small under the influence of cohort effects, and the contribution of the cohort effect to the changes in the incidence of asthma in the past three decades needs to be further discovered. However, the increased RR of incidence in the recent birth cohort should be of concern, indicating increased exposure to asthma risk factors. The success of action taken for asthma in Finland provided evidence that controlling asthma risk factors reduces the incidence of asthma. The Finnish Allergy Programme (2008–2018) introduced the concept of The Nature Step to Respiratory Health and led to actions relevant to society and healthcare as a whole (29, 30). In Finland, natural steps have been implemented in prevention of asthma: where (i) strengthening connexions with natural environments and increasing physical activity, (ii) increasing use of fresh vegetables and fruits and water, avoiding sugary drinks and consumption of tobacco and alcohol, (iii) linking with natural elements especially in the care of children and the elderly (31). In Finland, the burden of asthma has fallen, with less medication use and a decrease in the severity of asthma.

The RR of asthma mortality decreased with birth cohort, and cohort effects contributed to the decline in asthma mortality over the past three decades. According to the GBD 2019 study, high body mass index, exposure to occupational asthmagens, and smoking were key risk factors for asthma death among quantifiable risks (7). The proportion of deaths attributable to smoking and occupational asthmagens has decreased over the past three decades, while the proportion attributable to high body mass index has increased, and high body mass index has become the largest risk factor for asthma death. The combined attributable risk of the three risk factors continued to decline, which could explain, to some extent, the decline in the relative risk of asthma mortality. Although unlike smoking and occupational asthmagens, a high body mass index is not widely recognised as a modifiable risk factor, promoting a healthy diet and physical activity and incorporating weight loss into the daily life management of asthma patients is still necessary to reduce the RR of asthma mortality.

This study specifically presented comprehensive estimates of asthma incidence and mortality by age, sex, and region from 1990 to 2019 to help track global, regional, and national progress in asthma control. In addition, this study was the first analysis of age effects, period effects, and birth cohort effects for global asthma incidence and mortality using an APC-IE analysis to assess the impact of possible factors on asthma onset and death. There were also limitations in this study. First, the diagnosis of asthma relied on self-reported wheezing and physician diagnosis over the past 12 months, as no physiological measure was considered the gold standard. Thus, the measurement of asthma incidence and mortality was subject to the limitations of recall bias, access to health services, and the different interpretations of survey questions inherent in self-reported health measurements (28). Second, the GBD study itself had limitations. The GBD study results were affected by the quality of the population and disease data collected. There was a lack of asthma-related data in many countries, and there was a gap between the findings and the real situation in countries around the world, the details of which have been discussed elsewhere (2, 7, 8). Third, although age, period, and cohort effects were estimated in this study, our age-period-cohort analysis was based on the estimated cross-sectional data of GBD from 1990 to 2019, which was not a cohort study. Large cohort studies in different countries are needed to establish location and time-specific relative risks.

## Conclusion

In conclusion, global asthma incidence and mortality declined from 1990 to 2019. Regions with lower SDIs have higher age-standardised mortality rates for asthma and deserve attention and priority support for medical resources. The Global Initiative for Asthma 2022 provided more detailed guidance on the diagnosis and management of asthma for resource-poor areas, to help improve respiratory health (32). Based on the APC-IE analysis, the decline in asthma incidence was mainly attributed to age and period effects, and the decline in asthma mortality was mainly attributed to period and cohort effects. Notably, the RR of asthma incidence continues to rise in the recent birth cohort, suggesting that exposure to environmental risk factors may increase. Global action and careful implementation of asthma-related health policies can help to better control asthma, and the Finnish Programme shows the way to action.

## Data availability statement

The original contributions presented in the study are publicly available. This data can be found here: https://www.jianguoyun.com/p/DemQ6wQQyuv3Chir6toEIAA.

## Author contributions

YC conceived the study and drafted the manuscript. YC and SC collected and analysed the data. SH and XC commented on the data analysis and interpretation of results. WZ, ZL, and YW participated in the data preparation and provided important comments on the manuscript. YC and SH are the guarantors. All authors contributed to the article and approved the submitted version. The corresponding author attests that all listed authors meet authorship criteria and that no others meeting the criteria have been omitted.

## Funding

This project was supported by grants from the National Natural Science Foundation of China (81960618), the Science and Technology Plan Project of Jiangxi Province Health Commission (202211345), the Medical Fostering Foundation of Nanchang University (PY201903), and Postgraduate Innovation Special Fund of Jiangxi Province (YC2021-S188). The funders did not have any role in study design, data collection and analysis, manuscript preparation and decision to publish.

## Conflict of interest

The authors declare that the research was conducted in the absence of any commercial or financial relationships that could be construed as a potential conflict of interest.

## Publisher's note

All claims expressed in this article are solely those of the authors and do not necessarily represent those of their affiliated organizations, or those of the publisher, the editors and the reviewers. Any product that may be evaluated in this article, or claim that may be made by its manufacturer, is not guaranteed or endorsed by the publisher.
