# Supplementary material for: Global trends in the incidence and mortality of asthma from 1990 to 2019: An age-period-cohort analysis using the global burden of disease study 2019
Source: Front Public Health. 2022 Nov 22;10:1036674. doi: 10.3389/fpubh.2022.1036674 (PMC9723391; doi:10.3389/fpubh.2022.1036674)
Supplement: Supplementary file 1 [file Data_Sheet_1.PDF]

Supplementary Material

Global trends in the incidence and mortality of asthma from 1990 to 2019: an Age-Period-Cohort Analysis using the Global Burden of Disease Study 2019

The supplementary material includes detailed information of data source, 3 supplementary tables and 1 supplementary figure.

1 Detailed information of data source

1.1 Data source of asthma death outcome estimation

Data used to estimate asthma mortality included vital registration and surveillance data from the cause of death (COD) database. Verbal autopsy data were not included and were instead mapped to an overall chronic respiratory model(1).

The GBD 2019 criteria for outliers excluded data points that (a) were implausibly high or low relative to global or regional patterns, (b) substantially conflicted with established age or temporal patterns, or (c) significantly conflicted with other data sources conducted from the same locations or locations with similar characteristics (ie, Socio-demographic Index)(1).

1.2 Data source of asthma nonfatal health outcome estimation

The original data resources for estimating incidence included systematic reviews, survey data, and certain national claims data.

No systematic review of the literature was completed for GBD2017 and GBD 2019 cycle. The last full systemic review of the literature on Asthma was done for GBD 2016 by the Institute for Health Metrics and Evaluation (IHME)(1, 2). (Asthma [Title/Abstract] AND prevalence [Title/Abstract] AND "Cross-Sectional Studies" [MeSH Terms]) were the search strings used in PubMed, which were filtered by studies of humans published between January 2012 and November 2016(3). Data in literature matching GBD's case definitions were extracted and those that had definitions outside GBD's alternative case definitions were not included.

In GBD 2016， the survey data include the Survey of Health, Ageing and Retirement in Europe (SHARE), the Russian Ural Eye and Medical Study, the South Africa National Income Dynamics Study, the South Africa General Household Survey 2009, and the WHO Study on Global Ageing and Adult Health series (SAGE), among others(2). Surveys carried out as part of the International Study of Asthma and Allergies in Childhood (ISAAC) collaboration are the most important source of prevalence data in children(1-3)d. These survey data continue to be used in the GBD 2019 study.

In addition to claims data used in GBD 2017, GBD 2019 added new USA claims data for the years 2015 and 2016. GBD 2019 also added new data for Wave 7 of the English Longitudinal Study of Ageing (ELSA)(1).

1. GBD 2019 Diseases and Injuries Collaborators. Global Burden of 369 Diseases and Injuries in 204 Countries and Territories, 1990–2019: A Systematic Analysis for the Global Burden of Disease Study 2019. *Lancet.* (2020) 396:1204-22. doi: 10.1016/s0140-6736(20)30925-9.

2. GBD 2017 Disease and Injury Incidence and Prevalence Collaborators. Global, Regional, and National Incidence, Prevalence, and Years Lived with Disability for 354 Diseases and Injuries for 195 Countries and Territories, 1990-2017: A Systematic Analysis for the Global Burden of Disease Study 2017. *Lancet.* (2018) 392:1789-858. doi: 10.1016/s0140-6736(18)32279-7.

3. GBD 2016 Disease and Injury Incidence and Prevalence Collaborators. Global, Regional, and National Incidence, Prevalence, and Years Lived with Disability for 328 Diseases and Injuries for 195 Countries, 1990-2016: A Systematic Analysis for the Global Burden of Disease Study 2016. *Lancet*. (2017) 390:1211-59. doi: 10.1016/s0140-6736(17)32154-2.

2 Supplementary Figures and Tables

2.1 Supplementary Figures

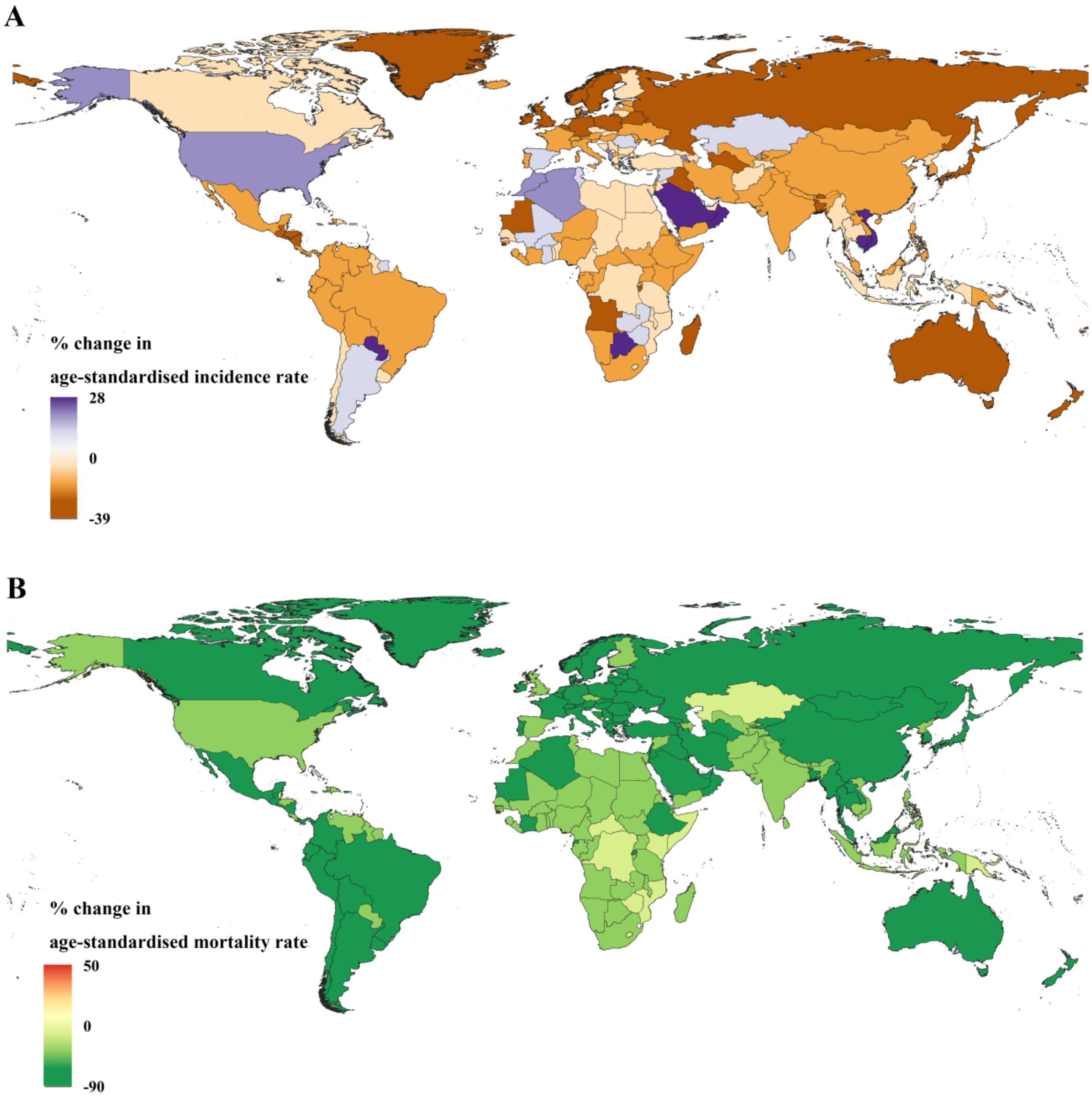

Supplementary Figure 1. The relative percentage change in the age-standardised incidence rate (A) and age-standardised mortality rate (B) of asthma between 1990 and 2019

2.2 Supplementary Tables

Supplementary Table 2. The SDI values by location in 1990 and 2019

Location	SDI Index Value,1990	SDI Index Value,2019	SDI Quintile,2019
Afghanistan	0.187	0.343	Low SDI
Albania	0.540	0.681	Middle SDI
Algeria	0.436	0.652	Middle SDI
American Samoa	0.606	0.712	High-middle SDI
Andorra	0.834	0.894	High SDI
Angola	0.238	0.470	Low-middle SDI
Antigua and Barbuda	0.579	0.743	High-middle SDI
Argentina	0.581	0.708	High-middle SDI
Armenia	0.536	0.689	Middle SDI
Australia	0.738	0.839	High SDI
Austria	0.753	0.849	High SDI
Azerbaijan	0.576	0.683	Middle SDI
Bahamas	0.692	0.796	High-middle SDI
Bahrain	0.553	0.751	High-middle SDI
Bangladesh	0.267	0.483	Low-middle SDI
Barbados	0.649	0.742	High-middle SDI
Belarus	0.591	0.745	High-middle SDI
Belgium	0.746	0.851	High SDI
Belize	0.428	0.603	Low-middle SDI
Benin	0.209	0.352	Low SDI
Bermuda	0.685	0.813	High SDI
Bhutan	0.228	0.455	Low-middle SDI
Bolivia (Plurinational State of)	0.412	0.566	Low-middle SDI
Bosnia and Herzegovina	0.533	0.718	High-middle SDI
Botswana	0.431	0.634	Middle SDI
Brazil	0.487	0.640	Middle SDI
Brunei Darussalam	0.676	0.823	High SDI
Bulgaria	0.631	0.764	High-middle SDI

Burkina Faso	0.125	0.257	Low SDI
Burundi	0.198	0.284	Low SDI
Cabo Verde	0.292	0.525	Low-middle SDI
Cambodia	0.266	0.469	Low-middle SDI
Cameroon	0.313	0.490	Low-middle SDI
Canada	0.790	0.873	High SDI
Central African Republic	0.186	0.274	Low SDI
Chad	0.108	0.238	Low SDI
Chile	0.592	0.759	High-middle SDI
China	0.433	0.686	Middle SDI
Colombia	0.478	0.633	Middle SDI
Comoros	0.274	0.455	Low SDI
Congo	0.364	0.568	Low-middle SDI
Cook Islands	0.625	0.764	High-middle SDI
Costa Rica	0.532	0.680	Middle SDI
Cote d'Ivoire	0.256	0.408	Low SDI
Croatia	0.680	0.794	High-middle SDI
Cuba	0.578	0.668	Middle SDI
Cyprus	0.662	0.841	High SDI
Czechia	0.688	0.828	High SDI
Democratic People's Republic of Korea	0.431	0.558	Low-middle SDI
Democratic Republic of the Congo	0.260	0.382	Low SDI
Denmark	0.806	0.890	High SDI
Djibouti	0.275	0.459	Low-middle SDI
Dominica	0.579	0.729	High-middle SDI
Dominican Republic	0.425	0.592	Low-middle SDI
Ecuador	0.503	0.640	Middle SDI
Egypt	0.403	0.658	Middle SDI
El Salvador	0.390	0.573	Low-middle SDI
Equatorial Guinea	0.208	0.685	Middle SDI
Eritrea	0.198	0.396	Low SDI
Estonia	0.665	0.835	High SDI
Eswatini	0.392	0.577	Low-middle SDI

Ethiopia	0.144	0.343	Low SDI
Fiji	0.527	0.664	Middle SDI
Finland	0.757	0.856	High SDI
France	0.738	0.834	High SDI
Gabon	0.388	0.656	Middle SDI
Gambia	0.218	0.399	Low SDI
Georgia	0.654	0.702	High-middle SDI
Germany	0.819	0.898	High SDI
Ghana	0.355	0.557	Low-middle SDI
Greece	0.682	0.794	High-middle SDI
Greenland	0.655	0.761	High-middle SDI
Grenada	0.463	0.669	Middle SDI
Guam	0.693	0.813	High SDI
Guatemala	0.315	0.526	Low-middle SDI
Guinea	0.175	0.325	Low SDI
Guinea-Bissau	0.200	0.355	Low SDI
Guyana	0.452	0.618	Middle SDI
Haiti	0.307	0.432	Low SDI
Honduras	0.330	0.496	Low-middle SDI
Hungary	0.659	0.791	High-middle SDI
Iceland	0.764	0.869	High SDI
India	0.327	0.566	Low-middle SDI
Indonesia	0.452	0.660	Middle SDI
Iran (Islamic Republic of)	0.404	0.670	Middle SDI
Iraq	0.392	0.671	Middle SDI
Ireland	0.730	0.867	High SDI
Israel	0.717	0.803	High-middle SDI
Italy	0.712	0.801	High-middle SDI
Jamaica	0.542	0.684	Middle SDI
Japan	0.791	0.870	High SDI
Jordan	0.520	0.731	High-middle SDI
Kazakhstan	0.602	0.723	High-middle SDI
Kenya	0.333	0.508	Low-middle SDI

Kiribati	0.425	0.527	Low-middle SDI
Kuwait	0.655	0.851	High SDI
Kyrgyzstan	0.532	0.596	Low-middle SDI
Lao People's Democratic Republic	0.268	0.490	Low-middle SDI
Latvia	0.675	0.820	High SDI
Lebanon	0.462	0.708	High-middle SDI
Lesotho	0.321	0.507	Low-middle SDI
Liberia	0.221	0.370	Low SDI
Libya	0.405	0.709	High-middle SDI
Lithuania	0.670	0.843	High SDI
Luxembourg	0.815	0.895	High SDI
Madagascar	0.265	0.396	Low SDI
Malawi	0.213	0.384	Low SDI
Malaysia	0.542	0.737	High-middle SDI
Maldives	0.303	0.562	Low-middle SDI
Mali	0.126	0.263	Low SDI
Malta	0.666	0.801	High-middle SDI
Marshall Islands	0.398	0.544	Low-middle SDI
Mauritania	0.308	0.496	Low-middle SDI
Mauritius	0.527	0.705	High-middle SDI
Mexico	0.507	0.649	Middle SDI
Micronesia (Federated States of)	0.447	0.580	Low-middle SDI
Monaco	0.834	0.902	High SDI
Mongolia	0.465	0.606	Low-middle SDI
Montenegro	0.701	0.791	High-middle SDI
Morocco	0.347	0.548	Low-middle SDI
Mozambique	0.120	0.307	Low SDI
Myanmar	0.284	0.521	Low-middle SDI
Namibia	0.454	0.612	Middle SDI
Nauru	0.499	0.618	Middle SDI

Nepal	0.198	0.422	Low SDI
Netherlands	0.796	0.883	High SDI
New Zealand	0.757	0.840	High SDI
Nicaragua	0.338	0.517	Low-middle SDI
Niger	0.073	0.162	Low SDI
Nigeria	0.305	0.515	Low-middle SDI
Niue	0.566	0.711	High-middle SDI
North Macedonia	0.618	0.744	High-middle SDI
Northern Mariana Islands	0.692	0.771	High-middle SDI
Norway	0.807	0.913	High SDI
Oman	0.441	0.783	High-middle SDI
Pakistan	0.247	0.449	Low SDI
Palau	0.621	0.738	High-middle SDI
Palestine	0.314	0.588	Low-middle SDI
Panama	0.544	0.686	Middle SDI
Papua New Guinea	0.292	0.394	Low SDI
Paraguay	0.465	0.638	Middle SDI
Peru	0.501	0.648	Middle SDI
Philippines	0.497	0.623	Middle SDI
Poland	0.632	0.802	High-middle SDI
Portugal	0.607	0.743	High-middle SDI
Puerto Rico	0.670	0.814	High SDI
Qatar	0.585	0.830	High SDI
Republic of Korea	0.686	0.878	High SDI
Republic of Moldova	0.585	0.696	High-middle SDI
Romania	0.625	0.760	High-middle SDI
Russian Federation	0.695	0.805	High-middle SDI
Rwanda	0.257	0.429	Low SDI
Saint Kitts and Nevis	0.583	0.746	High-middle SDI
Saint Lucia	0.483	0.670	Middle SDI
Saint Vincent and the Grenadines	0.462	0.627	Middle SDI
Samoa	0.531	0.641	Middle SDI
San Marino	0.814	0.884	High SDI

Sao Tome and Principe	0.299	0.502	Low-middle SDI
Saudi Arabia	0.480	0.805	High-middle SDI
Senegal	0.227	0.389	Low SDI
Serbia	0.626	0.767	High-middle SDI
Seychelles	0.567	0.724	High-middle SDI
Sierra Leone	0.207	0.347	Low SDI
Singapore	0.688	0.861	High SDI
Slovakia	0.656	0.812	High SDI
Slovenia	0.726	0.840	High SDI
Solomon Islands	0.279	0.407	Low SDI
Somalia	0.051	0.081	Low SDI
South Africa	0.552	0.678	Middle SDI
South Sudan	0.248	0.363	Low SDI
Spain	0.647	0.767	High-middle SDI
Sri Lanka	0.504	0.690	Middle SDI
Sudan	0.227	0.515	Low-middle SDI
Suriname	0.498	0.636	Middle SDI
Sweden	0.769	0.872	High SDI
Switzerland	0.868	0.929	High SDI
Syrian Arab Republic	0.367	0.619	Middle SDI
Taiwan (Province of China)	0.667	0.868	High SDI
Tajikistan	0.468	0.539	Low-middle SDI
Thailand	0.508	0.687	Middle SDI
Timor-Leste	0.274	0.514	Low-middle SDI
Togo	0.266	0.417	Low SDI
Tokelau	0.427	0.626	Middle SDI
Tonga	0.510	0.636	Middle SDI
Trinidad and Tobago	0.618	0.757	High-middle SDI
Tunisia	0.434	0.672	Middle SDI
Turkey	0.473	0.748	High-middle SDI
Turkmenistan	0.548	0.670	Middle SDI
Tuvalu	0.426	0.589	Low-middle SDI
Uganda	0.167	0.404	Low SDI
Ukraine	0.653	0.736	High-middle SDI

United Arab Emirates	0.621	0.880	High SDI
United Kingdom	0.745	0.847	High SDI
United Republic of Tanzania	0.260	0.423	Low SDI
United States of America	0.768	0.859	High SDI
United States Virgin Islands	0.667	0.799	High-middle SDI
Uruguay	0.581	0.697	High-middle SDI
Uzbekistan	0.490	0.631	Middle SDI
Vanuatu	0.361	0.485	Low-middle SDI
Venezuela (Bolivarian Republic of)	0.509	0.607	Low-middle SDI
Viet Nam	0.390	0.617	Middle SDI
Yemen	0.176	0.412	Low SDI
Zambia	0.299	0.505	Low-middle SDI
Zimbabwe	0.394	0.476	Low-middle SDI

Supplementary Table 2. Asthma incidence rates estimated coefficients for the age, period and cohort effects.

Factor	Coefficients (95%CI)		
	Both	Male	Female
Age			
0-4	1.14 (1.11 to 1.17)	1.28 (1.24 to 1.31)	1.00 (0.96 to 1.03)
5-9	0.70 (0.66 to 0.73)	0.87 (0.83 to 0.90)	0.52 (0.48 to 0.55)
10-14	0.25 (0.21 to 0.29)	0.28 (0.24 to 0.32)	0.24 (0.20 to 0.28)
15-19	-0.06 (-0.10 to -0.01)	-0.12 (-0.16 to -0.07)	0.01 (-0.03 to 0.05)
20-24	-0.32 (-0.37 to -0.27)	-0.42 (-0.47 to -0.36)	-0.24 (-0.28 to -0.19)
25-29	-0.58 (-0.63 to -0.52)	-0.69 (-0.75 to -0.63)	-0.47 (-0.52 to -0.42)
30-34	-0.68 (-0.74 to -0.62)	-0.82 (-0.88 to -0.75)	-0.56 (-0.62 to -0.51)
35-39	-0.57 (-0.63 to -0.51)	-0.71 (-0.78 to -0.65)	-0.45 (-0.50 to -0.39)
40-44	-0.48 (-0.54 to -0.43)	-0.60 (-0.66 to -0.54)	-0.38 (-0.43 to -0.32)

45-49	-0.43 (-0.49 to -0.37)	-0.50 (-0.56 to -0.44)	-0.36 (-0.41 to -0.31)
50-54	-0.24 (-0.3 to -0.19)	-0.25 (-0.3 to -0.20)	-0.23 (-0.28 to -0.18)
55-59	0.01 (-0.04 to 0.05)	0.05 (0.00 to 0.09)	-0.02 (-0.07 to 0.02)
60-64	0.15 (0.11 to 0.19)	0.23 (0.18 to 0.27)	0.10 (0.06 to 0.14)
65-69	0.24 (0.21 to 0.28)	0.33 (0.29 to 0.37)	0.17 (0.13 to 0.21)
70-74	0.17 (0.14 to 0.21)	0.26 (0.23 to 0.30)	0.10 (0.06 to 0.14)
75-79	-0.04 (-0.08 to 0.00)	0.07 (0.03 to 0.11)	-0.12 (-0.16 to -0.08)
80-84	-0.02 (-0.06 to 0.02)	0.06 (0.02 to 0.10)	-0.08 (-0.12 to -0.04)
85-89	0.25 (0.21 to 0.29)	0.25 (0.22 to 0.29)	0.24 (0.20 to 0.28)
90-94	0.51 (0.47 to 0.55)	0.44 (0.40 to 0.48)	0.53 (0.49 to 0.57)
Period			
1992	0.18 (0.16 to 0.20)	0.17 (0.15 to 0.19)	0.18 (0.16 to 0.20)
1997	0.08 (0.06 to 0.10)	0.08 (0.06 to 0.10)	0.08 (0.06 to 0.10)
2002	-0.02 (-0.04 to 0.00)	-0.02 (-0.04 to 0.00)	-0.02 (-0.04 to 0.00)
2007	-0.07 (-0.09 to -0.05)	-0.07 (-0.09 to -0.05)	-0.07 (-0.09 to -0.05)
2012	-0.08 (-0.10 to -0.06)	-0.08 (-0.10 to -0.06)	-0.09 (-0.11 to -0.07)
2017	-0.09 (-0.11 to -0.06)	-0.08 (-0.10 to -0.06)	-0.09 (-0.11 to -0.07)
Cohort			
1900-1904	-0.26 (-0.34 to -0.18)	-0.21 (-0.30 to -0.13)	-0.29 (-0.37 to -0.21)
1905-1909	-0.17 (-0.23 to -0.11)	-0.12 (-0.18 to -0.06)	-0.20 (-0.26 to -0.14)
1910-1914	-0.06 (-0.11 to -0.01)	-0.02 (-0.07 to 0.03)	-0.09 (-0.14 to -0.03)
1915-1919	0.07 (0.02 to 0.11)	0.09 (0.05 to 0.13)	0.05 (0.00 to 0.09)
1920-1924	0.13 (0.09 to 0.17)	0.15 (0.11 to 0.19)	0.11 (0.07 to 0.15)

1925-1929	0.14<br>(0.10 to 0.17)	0.16<br>(0.12 to 0.19)	0.12<br>(0.08 to 0.15)
1930-1934	0.13<br>(0.09 to 0.16)	0.13<br>(0.09 to 0.17)	0.12<br>(0.08 to 0.16)
1935-1939	0.08<br>(0.04 to 0.12)	0.06<br>(0.02 to 0.1)	0.09<br>(0.05 to 0.13)
1940-1944	0.02<br>(-0.02 to 0.06)	-0.01<br>(-0.05 to 0.03)	0.05<br>(0.00 to 0.09)
1945-1949	-0.04<br>(-0.08 to 0.01)	-0.07<br>(-0.12 to -0.02)	-0.01<br>(-0.05 to 0.04)
1950-1954	-0.09<br>(-0.14 to -0.04)	-0.13<br>(-0.18 to -0.08)	-0.06<br>(-0.11 to -0.01)
1955-1959	-0.13<br>(-0.18 to -0.07)	-0.16<br>(-0.22 to -0.10)	-0.1<br>(-0.15 to -0.04)
1960-1964	-0.11<br>(-0.17 to -0.05)	-0.14<br>(-0.21 to -0.08)	-0.09<br>(-0.14 to -0.03)
1965-1969	-0.16<br>(-0.22 to -0.10)	-0.17<br>(-0.24 to -0.11)	-0.15<br>(-0.21 to -0.09)
1970-1974	-0.14<br>(-0.20 to -0.08)	-0.15<br>(-0.21 to -0.08)	-0.14<br>(-0.19 to -0.08)
1975-1979	-0.10<br>(-0.16 to -0.05)	-0.11<br>(-0.16 to -0.05)	-0.10<br>(-0.15 to -0.05)
1980-1984	-0.06<br>(-0.11 to -0.01)	-0.07<br>(-0.13 to -0.02)	-0.05<br>(-0.10 to -0.01)
1985-1989	-0.05<br>(-0.09 to -0.01)	-0.06<br>(-0.10 to -0.02)	-0.04<br>(-0.08 to 0.00)
1990-1994	0.00<br>(-0.04 to 0.03)	0.00<br>(-0.04 to 0.03)	-0.01<br>(-0.04 to 0.03)
1995-1999	0.03<br>(0.00 to 0.07)	0.03<br>(0.00 to 0.07)	0.03<br>(0.00 to 0.07)
2000-2004	0.07<br>(0.04 to 0.11)	0.08<br>(0.04 to 0.12)	0.07<br>(0.03 to 0.11)
2005-2009	0.15<br>(0.10 to 0.19)	0.15<br>(0.11 to 0.19)	0.14<br>(0.10 to 0.18)
2010-2014	0.24<br>(0.20 to 0.28)	0.25<br>(0.20 to 0.29)	0.23<br>(0.18 to 0.27)
2015-2019	0.32<br>(0.26 to 0.38)	0.33<br>(0.27 to 0.39)	0.30<br>(0.24 to 0.36)

Supplementary Table 3. Asthma mortality rates estimated coefficients for the age, period and cohort effects.

Factor	Coefficients (95%CI)		
	Both	Male	Female
Age			
0-4	-0.28<br>(-1.02 to 0.46)	-0.39<br>(-1.13 to 0.36)	-0.19<br>(-0.93 to 0.55)
5-9	-1.82<br>(-2.96 to -0.69)	-1.90<br>(-3.04 to -0.76)	-1.75<br>(-2.89 to -0.62)
10-14	-1.93<br>(-3.04 to -0.81)	-1.92<br>(-3.00 to -0.84)	-1.96<br>(-3.11 to -0.80)
15-19	-1.71<br>(-2.67 to -0.75)	-1.80<br>(-2.78 to -0.82)	-1.63<br>(-2.57 to -0.69)
20-24	-1.48<br>(-2.32 to -0.64)	-1.51<br>(-2.34 to -0.67)	-1.47<br>(-2.32 to -0.63)
25-29	-1.34<br>(-2.11 to -0.56)	-1.36<br>(-2.14 to -0.59)	-1.32<br>(-2.10 to -0.55)
30-34	-1.16<br>(-1.87 to -0.44)	-1.23<br>(-1.97 to -0.50)	-1.10<br>(-1.79 to -0.4)
35-39	-0.89<br>(-1.51 to -0.26)	-0.94<br>(-1.58 to -0.30)	-0.84<br>(-1.46 to -0.23)
40-44	-0.59<br>(-1.13 to -0.05)	-0.62<br>(-1.16 to -0.07)	-0.57<br>(-1.10 to -0.04)
45-49	-0.23<br>(-0.68 to 0.22)	-0.24<br>(-0.70 to 0.22)	-0.23<br>(-0.68 to 0.22)
50-54	0.13<br>(-0.25 to 0.50)	0.17<br>(-0.21 to 0.54)	0.08<br>(-0.29 to 0.45)
55-59	0.48<br>(0.18 to 0.78)	0.52<br>(0.22 to 0.82)	0.44<br>(0.14 to 0.74)
60-64	0.85<br>(0.61 to 1.09)	0.90<br>(0.67 to 1.14)	0.79<br>(0.54 to 1.03)
65-69	1.11<br>(0.91 to 1.31)	1.19<br>(0.99 to 1.38)	1.02<br>(0.82 to 1.23)
70-74	1.40<br>(1.22 to 1.58)	1.45<br>(1.27 to 1.62)	1.36<br>(1.17 to 1.54)
75-79	1.64<br>(1.45 to 1.84)	1.70<br>(1.51 to 1.89)	1.60<br>(1.41 to 1.80)
80-84	1.89<br>(1.66 to 2.12)	1.91<br>(1.69 to 2.14)	1.88<br>(1.65 to 2.11)
85-89	1.95<br>(1.67 to 2.23)	2.01<br>(1.73 to 2.29)	1.93<br>(1.65 to 2.21)
90-94	1.97<br>(1.63 to 2.31)	2.07<br>(1.73 to 2.41)	1.96<br>(1.62 to 2.3)
Period			

1992	0.05 (-0.14 to 0.23)	0.04 (-0.14 to 0.23)	0.04 (-0.14 to 0.23)
1997	0.05 (-0.07 to 0.18)	0.07 (-0.05 to 0.19)	0.04 (-0.08 to 0.17)
2002	0.02 (-0.06 to 0.11)	0.03 (-0.05 to 0.11)	0.02 (-0.06 to 0.11)
2007	-0.02 (-0.11 to 0.07)	-0.02 (-0.1 to 0.07)	-0.02 (-0.11 to 0.07)
2012	-0.07 (-0.21 to 0.06)	-0.08 (-0.21 to 0.05)	-0.07 (-0.20 to 0.06)
2017	-0.03 (-0.22 to 0.16)	-0.05 (-0.23 to 0.14)	-0.02 (-0.21 to 0.16)
Cohort			
1900-1904	1.25 (0.82 to 1.69)	1.42 (0.99 to 1.85)	1.14 (0.71 to 1.58)
1905-1909	1.15 (0.80 to 1.51)	1.28 (0.93 to 1.64)	1.06 (0.70 to 1.42)
1910-1914	1.04 (0.75 to 1.33)	1.15 (0.86 to 1.44)	0.96 (0.67 to 1.25)
1915-1919	0.97 (0.74 to 1.2)	1.04 (0.82 to 1.27)	0.91 (0.68 to 1.14)
1920-1924	0.89 (0.71 to 1.07)	0.93 (0.76 to 1.11)	0.84 (0.66 to 1.02)
1925-1929	0.75 (0.60 to 0.90)	0.78 (0.63 to 0.92)	0.72 (0.57 to 0.87)
1930-1934	0.66 (0.50 to 0.81)	0.64 (0.49 to 0.79)	0.65 (0.49 to 0.8)
1935-1939	0.57 (0.38 to 0.76)	0.52 (0.33 to 0.70)	0.60 (0.41 to 0.79)
1940-1944	0.43 (0.18 to 0.67)	0.36 (0.12 to 0.60)	0.48 (0.23 to 0.72)
1945-1949	0.32 (0.01 to 0.63)	0.24 (-0.07 to 0.55)	0.39 (0.08 to 0.7)
1950-1954	0.12 (-0.27 to 0.50)	0.04 (-0.35 to 0.42)	0.19 (-0.20 to 0.58)
1955-1959	-0.01 (-0.47 to 0.45)	-0.08 (-0.54 to 0.39)	0.05 (-0.41 to 0.51)
1960-1964	-0.07 (-0.62 to 0.47)	-0.15 (-0.69 to 0.40)	-0.01 (-0.55 to 0.53)
1965-1969	-0.20 (-0.83 to 0.42)	-0.29 (-0.93 to 0.34)	-0.12 (-0.74 to 0.50)
1970-1974	-0.30 (-1.00 to 0.41)	-0.35 (-1.07 to 0.36)	-0.24 (-0.94 to 0.46)
1975-1979	-0.34 (-1.12 to 0.44)	-0.38 (-1.17 to 0.40)	-0.30 (-1.08 to 0.47)

1980-1984	-0.44 (-1.31 to 0.42)	-0.46 (-1.33 to 0.40)	-0.43 (-1.29 to 0.43)
1985-1989	-0.57 (-1.51 to 0.37)	-0.57 (-1.51 to 0.37)	-0.57 (-1.51 to 0.38)
1990-1994	-0.63 (-1.44 to 0.18)	-0.62 (-1.43 to 0.19)	-0.64 (-1.45 to 0.17)
1995-1999	-0.78 (-1.71 to 0.15)	-0.77 (-1.70 to 0.16)	-0.79 (-1.72 to 0.14)
2000-2004	-0.93 (-2.01 to 0.15)	-0.92 (-2.01 to 0.17)	-0.95 (-2.02 to 0.13)
2005-2009	-1.08 (-2.33 to 0.17)	-1.07 (-2.33 to 0.19)	-1.09 (-2.34 to 0.15)
2010-2014	-1.26 (-2.72 to 0.20)	-1.24 (-2.72 to 0.24)	-1.27 (-2.71 to 0.17)
2015-2019	-1.54 (-3.48 to 0.41)	-1.49 (-3.46 to 0.47)	-1.57 (-3.49 to 0.35)
